# Services and staffing practices in academic health sciences libraries serving college of osteopathic medicine programs: a mixed methods study

**DOI:** 10.5195/jmla.2020.862

**Published:** 2020-07-01

**Authors:** Joanne M. Muellenbach, Wendy C. Duncan, Cheryl Vanier, Lisa A. Ennis, Anna Yang

**Affiliations:** 1 jmuellenbach@chsu.edu, Director, Health Sciences Library, and Associate Professor, California Health Sciences University, Clovis, CA; 2 wduncan@chsu.edu, Senior Vice-President for Academic Affairs and Provost, California Health Sciences University, Clovis, CA; 3 cheryl.vanier@tun.touro.edu, Chief Research Officer, Touro University Nevada, Henderson, NV; 4 lennis@acom.edu, Director, Library & Learning Resources, Alabama College of Osteopathic Medicine, Dothan, AL; 5 ayang3@scu.edu, Science Librarian, Santa Clara University, Santa Clara, CA

## Abstract

**Objective:**

This study describes and assesses services, staffing practices, and trends in academic health sciences libraries that serve accredited college of osteopathic medicine (COM) programs in the United States.

**Methods:**

The study was conducted in three phases. In phase one, the investigators collected data on library services and staffing through the publicly facing websites of the COM libraries. In phase two, thirty-five COM library directors were invited to complete a survey regarding their services, staffing, supported programs, and students served. In phase three, seven COM library directors participated in phone interviews regarding services that increased their visibility, their motivation to offer expanded services, adequacy of staffing, and competencies required for new librarian roles. The investigators incorporated the Medical Library Association (MLA) competencies as a framework to structure the results.

**Results:**

Phase one identified 35 COM libraries serving between 162 and 8,281 students. In phase two, 30 out of a possible 35 survey respondents indicated that the top services offered or considered by COM libraries were in the MLA competency areas of “Instruction & Instructional Design” and “Evidence-Based Practice & Research.” In addition, we discovered that COM libraries had a median of 10 full-time equivalent (FTE) staff per 1,000 students. Phase three data revealed that library directors attributed their libraries' success to the skills and talents of their staff, the wide range of resources and services they offered, and the desirability of their physical spaces. Library directors identified skills in the same MLA competency areas as phase two, as well as in the MLA competency areas of “Information Management” and “Leadership & Management,” as being desirable for new staff.

**Conclusion:**

The study results provide information for medical school administrators and library directors to help identify trends across US osteopathic medical schools in order to justify the need for additional services and staffing. These results can assist medical and library leadership in COM schools in planning for their future academic health sciences libraries. Finally, the findings could assist programs in library and information sciences in redesigning their curriculums based on the MLA competencies for students who seek future careers in academic health sciences libraries.

## INTRODUCTION

Academic health sciences libraries provide evidence-based, scholarly information to support education, research, and patient care. Guidance for such libraries has been provided by the Medical Library Association (MLA) Task Force to Review MLA's Competencies for Lifelong Learning and Professional Success, which has identified six competencies necessary for the professional success of health sciences librarians [1]. The MLA competencies are (1) “Information Services,” (2) “Information Management,” (3) “Instruction & Instructional Design,” (4) “Leadership & Management,” (5) “Evidence-Based Practice & Research,” and (6) “Health Information Professionalism.” Definitions and performance indicators are provided for achievement at the basic and expert levels for each competency.

Given the technological advances in the twenty-first century and the focus of academic health sciences libraries on providing 24/7 seamless access to online resources, library leaders need staff who possess the competencies to support evolving digital libraries. A perspective by Lindberg and Humphreys on the future of medical libraries predicted that, by 2015, “despite ubiquitous access to electronic information and the availability of multimedia digital libraries, digital libraries still need librarians” [2]. McClure, in her introduction to a special issue of the *Journal of the Medical Library Association (JMLA)* on new roles for health sciences librarians, remarked that since the 1970s, it was the traditional librarian who has had “the drive and energy to take on this new challenge [and] who has become the librarian of today” [3].

While there was an absence of research that reported on the current practices of academic health sciences libraries serving college of osteopathic medicine (COM) programs, a paper by Clarke and Thomas noted that librarians working in health areas have acquired a *specialist* status and are increasingly involved in such areas as critical appraisal of the journal literature, grading of the evidence, and study methodologies [4]. To address the needs of new health librarian “trainees,” Clarke and colleagues developed a Health Library Staff Competency Framework, based on Lave and Wegner's “Legitimate Peripheral Participation” (LPP) model, whereby trainees move toward being full members of a health librarian “community of practice” [5]. A study conducted in Ireland by Lawton and Burns identified ten competency areas in common with three of five library associations and concluded that once competencies are identified and adopted, librarians can create professional development plans to ensure their skills are current [6]. An article by Epstein, who at the time was MLA president, reported on the revised MLA competencies and the evolving roles of health sciences librarians in supporting bioinformatics and research data management [7].

A paper by Fitterling and colleagues highlighted yet another emerging role: developer of the medical information literacy Q-Bank, which was a collaboration amongst three COM institutions [8]. A scoping review by Ma and colleagues identified the emerging roles of health information professionals and reported that these “embedded librarians” have adopted roles in such areas as informatics collaboration, liaison, outreach, inclusion, and patient support and advocacy [9]. As a result, they created a new model that linked these roles to the MLA competencies. A special paper by Dexter and colleagues further suggested that, for newly established medical schools, libraries should tailor the resources and services offered to local conditions, including the needs of the community, the vision of the medical schools that they support, and the medical school curriculum [10].

COM programs have experienced rapid growth in medical education. The Liaison Committee on Medical Education [11] and the American Osteopathic Association [12] data reported that twenty-nine new allopathic and twenty-three new osteopathic medical schools received initial accreditation between 2000 and 2019. While osteopathic and allopathic medical programs are similar in producing highly skilled physicians, a study by Peters and colleagues has suggested that osteopathic medicine takes a more holistic approach to the patient, focuses on preventive medicine, and focuses on producing primary care physicians [13]. However, a new single accreditation system further narrows the differences between medical doctors and doctors of osteopathy as all residents and fellows will have to meet the same training standards [14].

This study evolved following a query from a provost whose university was developing a new COM. The university provost, also a member of the study team, sought guidance regarding the competencies that future health sciences librarians need. A literature search revealed a knowledge gap in this area. Given that the study investigators were employed at COM institutions in administration, the health sciences library, and research, COM libraries became the focus of our study, and two research questions emerged:

1. What are the services being offered or considered by COM libraries and how can they be mapped to the MLA competency framework?
2. What are the competencies, skills, and motivating factors that COM librarians need to succeed in these new roles to increase the value and visibility of their libraries?

The study objectives were to identify eligible COM libraries, survey COM library directors, and interview selected COM library directors about current and future services, staffing models, and preferred staff competencies. The results would assist medical and library leadership in COM programs in planning and transforming their libraries.

## METHODS

We conducted a three-phase study, based on a design used by Eldredge and colleagues in an analysis of library and informatics training practices at US medical schools [15]. The study design methods included an analysis of COM library websites, a survey, and telephone interviews.

### Phase one

In September 2018, the lead investigator consulted the list of medical schools from the American Association of Colleges of Osteopathic Medicine website [16] to identify all US COM libraries. The environmental scan of COM libraries' publicly facing websites initially started with collecting the library directors' contact details on an Excel spreadsheet, but after further review and reflection, we added columns with information about innovative services that the libraries offered, number and types of staff, and availability of library annual reports, course guides, and newsletters.

### Phase two

Through a literature review, we identified annual surveys developed by the Association of Academic Health Sciences Libraries (AAHSL) [17], including the AAHSL Services and Resources Survey Instrument, which informed the design of our survey (supplemental Appendix A). The survey objective was to identify innovative and unique library services that went beyond traditional services such as collection development, document delivery, reference, and literature search assistance. Survey questions asked about current services as well as services being considered for implementation within the fiscal year.

We organized the first 15 survey questions in MLA competency categories: Information Services (1 question), Information Management (2 questions), Instruction & Instructional Design (3 questions), Leadership & Management (1 question), Evidence-Based Practice & Research (4 questions), and Health Information Professionalism (4 questions). Questions about staffing practices—including types and numbers, additional programs that the COM libraries supported, and the total number of students served—were also included. Our data analysis used library full-time equivalent (FTE) per 1,000 students, a standard measure [18]. On October 9, 2018, the lead investigator distributed the survey by email, in the body of the message and as an attachment, to the library directors at each of the 35 main library locations identified in phase one. Two reminders were sent on October 15 and October 20. We used descriptive statistics to evaluate the survey results.

### Phase three

In phase three, twelve COM library directors were contacted, and seven accepted our invitation to participate in thirty-minute telephone interviews. We selected library directors from institutions that were established from 2000 to the present and, based on their data from phase two, indicated that they had experience developing new medical school libraries. The lead investigator conducted the telephone interviews, which consisted of five in-depth questions related to services that increased the library's visibility, the extent to which their librarians integrated information literacy and evidence-based medicine into the curriculum, their motivation to offer new services, the adequacy of current staffing levels, and the competencies needed for serving in these new roles (supplemental Appendix B).

Responses from the telephone interviews were captured by hand and entered into an Excel spreadsheet. Then, an analysis of the responses was performed by the research analyst and confirmed by the lead investigator to identify themes and to determine which themes aligned with the specific MLA competencies. Touro University Nevada's Institutional Review Board classified the study as not human subjects research (TUNIRB000038).

## RESULTS

### Phase one: Identification of library directors via websites

Thirty-five COM libraries were identified. While many of the COM library websites were easy to locate and provided useful information, some were difficult to find and lacking in detail.

### Phase two: Survey of library directors

**Figure 1:**
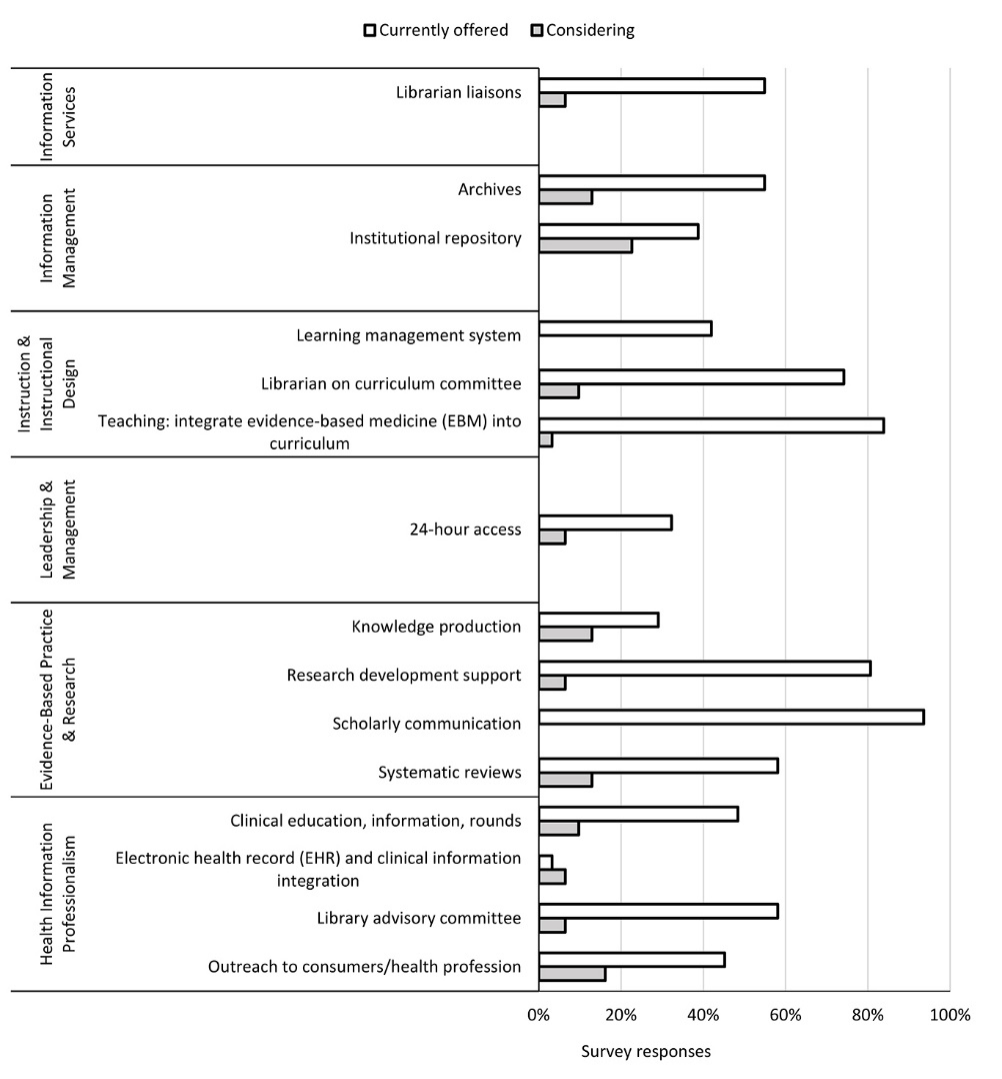
Summary of library services offered and being considered by the responding library directors, grouped by Medical Library Association (MLA) competencies

In phase two, 30 out of a possible 35 library directors completed the survey, for an 86% response rate. The top 5 services that COM libraries currently offered were activities associated with MLA competency 3, Instruction & Instructional Design, and MLA competency 5, Evidence-Based Practice & Research (Figure 1). The services that were most frequently mentioned as future offerings were in MLA competency 2, Information Management, and MLA competency 6, Health Information Professionalism. Respondents provided examples of additional services that their libraries offered that fit into MLA competency 1, Information Services (support to adjunct faculty, embedded librarians in the osteopathic manipulative medicine department, and support to alumni and residents); MLA competency 3, Instruction & Instructional Design (serving as course director and teaching medical informatics, information literacy, and research methods courses); MLA competency 4, Leadership & Management (providing a study hall); MLA competency 5, Evidence-Based Practice & Research (resume and writing assistance, poster printing, research methods, and thesis development); and MLA competency 6, Health Information Professionalism (outreach to high school students and collaboration with departments in the marketing and development of programs).

**Figure 2:**
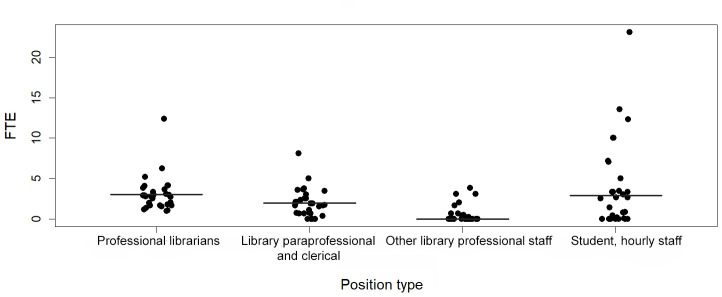
Median (lines) and individual (dots) library staffing per 1,000 students by employee position type in COM libraries

COM libraries varied in the types and number of staff they employed, but the variation is best understood relative to the number of students served by each library, which ranged from 162 to 8,281, with a median of 1,200 students (Table 1). Considering professional librarians, other professional library staff, paraprofessionals, and library student workers together, the median library FTEs per 1,000 students was 10, with a range of 2.1 to 30.8. The libraries serving the largest student populations tended to have smaller FTEs per 1,000 students than libraries serving the smallest student populations. Libraries also varied in their numbers of different types of staff per 1,000 students, particularly concerning students or hourly staff and librarians (Figure 2). Overall, the median for professional librarians and library student workers was 3 FTEs.

**Table 1:** Number of students served, total full-time equivalent (FTE) library student or hourly staff, and FTE per 1,000 students

Number of students served	Total full-time equivalent (FTE) library student or hourly staff	FTE per 1,000 students
162	2.0	12.3
260	8.0	30.8
369	9.0	24.4
492	4.5	9.1
570	3.2	5.6
600	4.0	6.7
600	5.0	8.3
648	3.1	4.8
650	11.0	16.9
800	7.4	9.3
852	9.0	10.6
965	10.0	10.4
1,100	10.8	9.8
1,200	11.3	9.4
1,200	23.0	19.2
1,400	12.5	8.9
1,435	8.8	6.1
1,477	5.2	3.5
1,500	8.0	5.3
1,549	12.7	8.2
1,549	12.7	8.2
1,800	15.0	8.3
2,000	31.0	15.5
2,031	6.0	3.0
2,300	35.0	15.2
2,860	20.0	7.0
3,560	12.5	3.5
5,600	30.0	5.4
8,000	16.5	2.1
8,281	21.4	2.6

**Figure 3:**
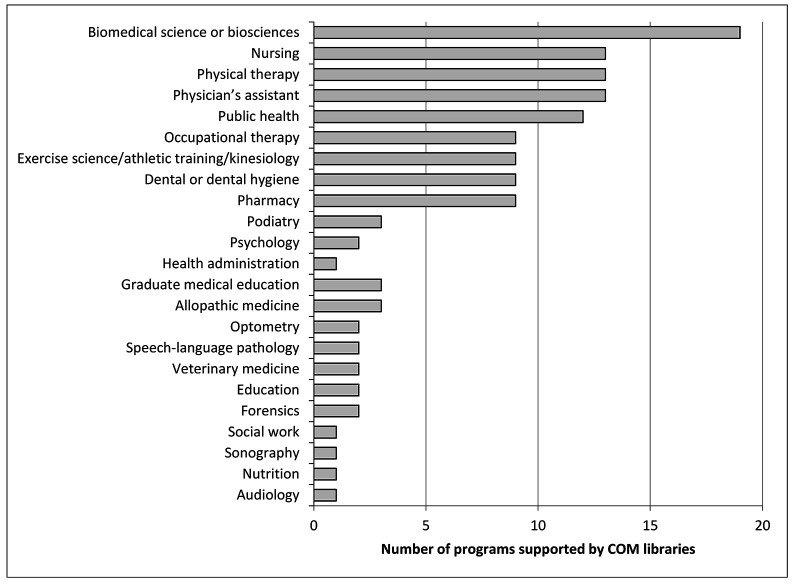
Additional programs beyond doctor of osteopathy supported by COM libraries

All libraries supported osteopathic medical schools, and twenty-one libraries supported additional programs (Figure 3).

### Phase three: In-depth telephone interviews

We conducted seven telephone interviews with library directors in November 2018, and four MLA competencies were identified as being relevant to the development of new COM libraries. While the answers varied, some recurring themes emerged from the responses to the questions.

**Figure 4:**
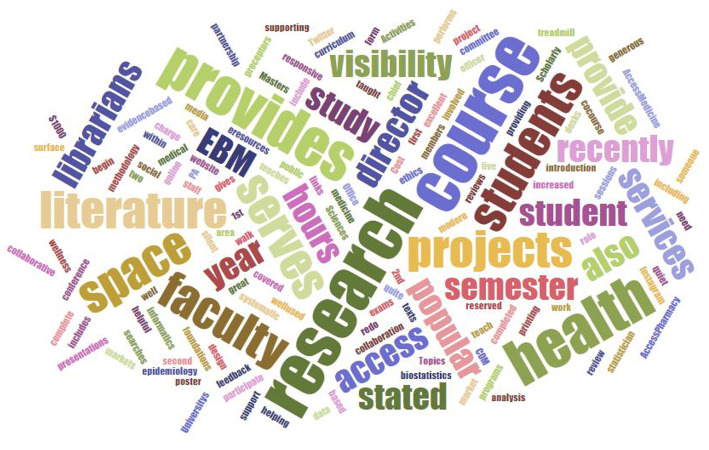
Wordle cloud from phase three interviews, question 1

**1. Of the services that your library is offering, which services have had the most success in increasing the visibility and value of the library?** Figure 4 presents a summary of the most frequently used words from this question. The services that increased the libraries' visibility included librarians serving on curriculum committees; teaching evidence-based medicine, research methods, and study design; and providing support on systematic reviews. One of the libraries provided a poster printing service that served to encourage collaboration in other areas of research. Finally, libraries increased their value by promoting their services on social media and by providing stand-up and treadmill desks, and collaborative and silent space.

**2. Could you explain the reasons for the success that you have experienced in integrating information literacy and evidence-based medicine in the medical school curriculum?** Reasons for the libraries' success were focused on the themes of reporting to deans or provosts who were encouraging and supportive. Library directors also explained that the reasons for their success were due to their librarians who served as course chairs, taught information literacy and evidence-based medicine skills, and were acknowledged as content experts. Finally, libraries achieved success by receiving positive course evaluations and through involvement in curricular research initiatives.

**3. What do you think motivates you and your librarians to move beyond the traditional services and offer new services?** Motivations for librarians to offer new services included enjoying the opportunities to be innovative, relevant, and visible in their institutions. Directors reported that librarians found it exciting and fulfilling to meet with students and faculty to help them utilize research tools and conduct systematic reviews. The results showed that librarians had a collaborative and inquisitive nature and found meaning in using their expertise to further student success. Library directors identified both internal and external motivators for moving beyond traditional library services. Internal motivators included the library's mission, the faculty and staff evaluation process, and innovative librarians with a desire to be involved in campus and community events. External motivators included direct requests from students and faculty and the high expectations of students.

**4. Reviewing the responses to the services offered (in phase two), do you feel that you have sufficient staff (types and numbers)?** The library directors acknowledged the need to hire additional professional librarians and staff. They stressed needing to not overextend themselves and commented that, when staff was out of the office, it was stressful to cover the full range of services. Interviewees remarked that many university administrators did not always have a deep understanding of the wide range of library services that were offered and the librarian competencies that were needed to deliver them. Library directors stated the importance of being prepared to make a strong case for hiring library personnel, especially when the COM was the only program that the library supported and the student FTE was small.

**Figure 5:**
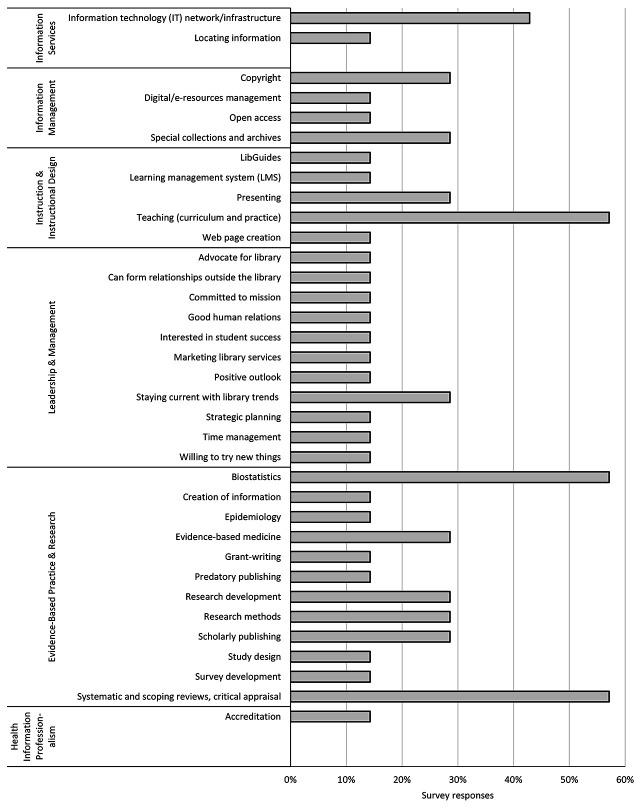
Summary of competencies and skills desired by COM library leaders

**5. Reviewing your responses to the services offered, what competencies and skills are needed by librarians and other professional staff to serve in these new roles?** Library directors highlighted necessary skills for librarians in each MLA competency category (Figure 5). In the area of Information Management (MLA competency 2), hiring professional librarians and staff with skills in electronic and technical services, library guide development, learning management systems (LMSs), and presentation software was considered desirable. The frequently mentioned librarian skills that were related to Instruction & Instructional Design (MLA competency 3) were serving as course directors, participating on curriculum committees, and providing program-specific library instruction. Interviewees also stressed the importance of using class assessments, holding faculty appointments, and being viewed as content experts. Interviewees identified librarian competencies needed in the area of Leadership & Management (MLA competency 4), such as promoting the library on social media. The competencies needed in the area of Evidence-Based Practice & Research (MLA competency 5) included skills in critical appraisal of the literature, teaching of biostatistics and epidemiology, and research development support.

## DISCUSSION

### Phase one

In phase one, we learned that the COM libraries' websites were not always reliable for gathering the data needed for our study. For example, some websites clearly showcased their programs and services, while others provided minimal information or were difficult to find. In fact, two of the library directors who were interviewed revealed that they served as course directors or co-taught in course programs, but this information was not found on their websites. Publicizing teaching roles could illustrate the depth of librarians' involvement in programs beyond the library walls. To facilitate comparisons across institutions, COM programs should consider how their public-facing sites provide information about library services and staffing practices.

### Phase two

Our survey found that the top two services being offered or considered by COM programs were in MLA competency 5, Evidence-Based Practice & Research (scholarly communication, research development support, systematic reviews, and knowledge production), and MLA competency 3, Instruction & Instructional Design (teaching and integration of EBM in the curriculum, librarian on the curriculum committee, and LMS). In MLA competency 3, having a librarian on the curriculum committee is critical. An article by Muellenbach and colleagues emphasized the numerous benefits of being members of course planning committees, including being provided with accounts for the LMSs and the opportunity to become deeply involved in the curriculum development process [19]. Having an LMS account also facilitated the librarians' ability to review course syllabi and link materials, such as library course guides, from the course sites. Overall, librarian involvement in teaching, research development, and support were the unifying themes that were identified as being key to the current and future success of the library.

A study by Hoover noted that because modern academic libraries are largely online, librarians and other professional staff are needed to serve in areas related to MLA competency 2, Information Management, to manage access to resources, use online tools for reference and research, and design websites [20]. In fact, it is becoming more common for the library space to be a hub for interaction with new technologies, such as 3D printers and virtual reality, and for data visualization. These library functions often require librarian support, indicating the need for a shift from library staff and student workers toward an increase in librarians or other professional staff for these specialized services. Although each of the responding COM libraries employ at least one professional librarian, our results related to the prevalence of innovative services highlight the need for COM libraries to focus on recruiting librarians and other professional staff as a part of their overall strategic planning.

The phase two survey identified a tendency for lower FTE staff numbers per 1,000 students as the number of students increased. Regardless of the number of students at an institution, most libraries initially require a certain number of librarians to provide a range of essential services. As student numbers increase, the core FTE librarians continue to offer a range of services to a larger student population. To address the increasing student FTEs, librarians should continue to incorporate creative solutions such as inviting faculty colleagues to serve as facilitators or moderators for their classroom activities or creating library research guides, tutorials, and videos that highlight the essential and in-demand library services.

Overall, we found a median of three FTEs for professional librarians and library student workers alike. While the relatively high number of student workers was surprising, it could be based on the need to staff the library in the evenings and on weekends. To address the need for additional professional librarians who can deliver the range of MLA competencies and serve in new and emerging roles, library directors may wish to redirect budget dollars that are spent on library student workers to hiring additional librarians. Possible alternatives to paying library student workers to extend the library's staffed hours could include the expanded use of self-service automated circulation systems, security guards, video camera monitoring, library science interns, and work study students.

### Phase three

The phase three interviews highlight the advantage of identifying champions amongst the teaching faculty and having the support of the curriculum administrators and coordinators. The development of relationships between the librarians and teaching faculty are critical, especially when teaching faculty must balance an already demanding curriculum with the students' growing need to be information literate. In the absence of a context or perceived need among the students, interview respondents report that stand-alone or one-shot library orientation sessions tend to be less well received. The investigators' experiences also suggest that the integration of library instruction into the curriculum results in improved educational outcomes.

COM library directors stressed the need to be strong and tireless advocates for library services, utilizing the skills in MLA competency 4, Leadership & Management, to ensure appropriate funding and staff support. In addition, to ensure the continued success of the library, library directors must have excellent interpersonal and communication skills, and the ability to mentor and provide staff with ongoing professional development opportunities. Phase three library directors were assertive and creative in designing and implementing new services, but they were also careful to assess, improve, and prioritize, so as not to spread the library staff too thin, in agreement with a study by Fought and Mitsunori on effective library leaders [21]. Fought and Mitsunori found that such leaders continually used data to make their cases, stayed current with trends, were effective recruiters and team-builders, and provided sufficient professional development opportunities to improve staff performance.

COM library directors largely attribute their libraries' accomplishments to the skills and talents of their staff. In fact, the importance attributed to library personnel suggests that hiring decisions may be among the most important decisions that library directors make. Based on the phase two survey results, by recruiting or providing training supporting MLA competency 5, Evidence-Based Practice & Research, and MLA competency 3, Instruction & Instructional Design, new COM librarians can make the greatest impact. Furthermore, the phase three results identify the MLA competencies of Health Information Professionalism and Leadership & Management as areas of future growth. The study results suggest that these MLA competencies should be a major focus when making hiring and training decisions.

### Limitations

This study has several weaknesses. The results represent the library directors' point of view, and the inclusion of other library personnel could have yielded a richer picture regarding future directions and reasons for success. In addition, this study targeted COM libraries. Future work might take a comparative approach by targeting other programs to get a more complex view of how libraries are shaped by the programs they serve. The interviews focused on libraries that were established from 2000 to the present, but older, well-established libraries could have contributed useful information as well. Finally, while the survey and interview questions identified some of the ways that libraries are collaborating with other departments on their campuses, it would be useful to highlight this topic in future research studies.

## CONCLUSION

This study provides an examination of current services and staffing practices in academic health sciences libraries serving COM programs in the United States. Library directors affiliated with COM programs can use these data to make a compelling case for appropriate budgets, space, and staffing. COM administrators and teaching faculty may find opportunities in the study's results for collaborations with the library, in such areas as curriculum-integrated instruction, research development, and scholarly publishing. We also hope that the study results will inspire program leaders in library and information science to design curricular content that focuses on all of the MLA competencies—particularly in the areas of Evidence-Based Practice & Research, Instruction & Instructional Design, Information Management, and Leadership & Management—to meet the needs of prospective students who are interested in careers as academic health sciences librarians. Brodman, who in 1965 served as MLA president, provided the following closing remarks in her presidential address, “Money Talks, but People Count,” at the sixty-fourth annual meeting of the MLA in Philadelphia:

I, therefore, address myself to my chronological peer group, those of us who are chiefs in our departments and libraries, and to those younger members who will be chiefs in the next few years. It is we who must bring forth the potentialities of our assistants, “and by potentialities I mean not just skills, but the full range…the capacities for sensing, wondering, learning, understanding, loving, and aspiring” [22]. It is we who day by day recruit for the future of our profession and whose personalities and abilities will determine whether or not medical librarianship is to include a large number of the best people in our society. It is we who will decide whether the fascination and the wonder of our calling will bring to our side those who can tackle with ability, enthusiasm, imagination, and the admiration of all observers the problems which, because of time and the bounds of human energy, we must necessarily leave unfinished. If we possess the hearing ear and the seeing eye, if the great humanistic goals of medical librarianship are ours, we need not worry about the future of our profession. For, although money talks, it is everlastingly true that it is the people who really count. [23]

## Data Availability

Data associated with this article are available through the Open Science Framework (OSF) at https://osf.io/kv9j3/.

## SUPPLEMENTAL FILES

**Appendix A:** College of osteopathic medicine services and staffing survey

**Appendix B:** Phase three: summarized responses to interview questions
