# Supplementary material for: Services and staffing practices in academic health sciences libraries serving college of osteopathic medicine programs: a mixed methods study
Source: J Med Libr Assoc. 2020 Jul 1;108(3):408–19. doi: 10.5195/jmla.2020.862 (PMC7441916; doi:10.5195/jmla.2020.862)
Supplement: Supplementary file 1 — Appendix A: College of osteopathic medicine services and staffing survey [file jmla-108-3-408-s01.pdf]

JMLA Journal of the Medical Library Association

SC
Supplemental content to
J Med Libr Assoc. Jul;108(3):dx.doi.org/10.5195/jmla.2020.862
www.jmla.mlanet.org
© Muellenbach, Duncan, Vanier, Ennis, Yang 2020

Services and staffing practices in academic health sciences libraries serving college of osteopathic medicine programs: a mixed methods study

Joanne M. Muellenbach, AHIP; Wendy C. Duncan; Cheryl Vanier; Lisa A. Ennis; Anna Yang

APPENDIX A

College of osteopathic medicine services and staffing survey

As the leader of a college of osteopathic (COM) library, you are invited to participate in a survey, to identify offered library services that go beyond the usual collection development, document delivery, reference, and literature search assistance. Additionally, it would be helpful to know your staff size. Survey results will be summarized and shared, so that COM libraries will have access to best practices to support accreditation, budgeting, and strategic planning.

Instructions: For each service, select: Yes; No; or Considering for this fiscal year.

1. Services offered	Yes	No	Considering
a. 24-hour access to library space			
b. Archives			
c. Clinical education/information/rounding			
d. Electronic health records and clinical information integration			
e. Institutional repository			
f. Knowledge production (3D printing/scanning, virtual reality, video production, and more)			
g. Learning management system support			
h. Library advisory committee			
i. Librarian liaisons (individual program support)			
j. Librarian membership on curriculum committees			
k. Outreach to consumers/health professionals			
l. Research development support			

JMLA Journal of the Medical Library Association

SC

Supplemental content to
J Med Libr Assoc. Jul;108(3):dx.doi.org/10.5195/jmla.2020.862
www.jmla.mlanet.org
© Muellenbach, Duncan, Vanier, Ennis, Yang 2020

1. Services offered	Yes	No	Considering
m. Scholarly communication (copyright, fair use, and more)			
n. Systematic reviews			
o. Teaching (integration of evidence-based practice within the curricula)			

Other services offered: ____________________

Instructions: For each staff type, please provide the **# of full-time equivalents (FTEs).**

2. Staffing provided	# of FTEs
a. Professional librarian faculty and staff	
b. Other library professional staff	
c. Library paraprofessionals and clerical staff	
d. Student, hourly, or non-permanent library assistant staff	
e. Total (all library faculty and staff)	

3. Demographics:

Name (First/Last): ________ Title: ________

Library: ________ Institution: ________

City: ________ State: ________

Telephone: ________ Email: ________

Programs supported by the library:

COM _____ Dental _____ School of nursing (SoN) _____ Physician assistant (PA) studies _____

Doctor of pharmacy (PharmD) _____ Occupational therapy (OT) _____ Physical therapy (PT) _____

Other programs: ____________________

Total # of students served:

Please return the completed survey to…
