# Supplementary material for: Services and staffing practices in academic health sciences libraries serving college of osteopathic medicine programs: a mixed methods study
Source: J Med Libr Assoc. 2020 Jul 1;108(3):408–19. doi: 10.5195/jmla.2020.862 (PMC7441916; doi:10.5195/jmla.2020.862)
Supplement: Supplementary file 2 — Appendix B: Phase three: summarized responses to interview questions [file jmla-108-3-408-s02.pdf]

JMLA Journal of the Medical Library Association

SC

Supplemental content to
J Med Libr Assoc. Jul;108(3):dx.doi.org/10.5195/jmla.2020.862
www.jmla.mlanet.org
© Muellenbach, Duncan, Vanier, Ennis, Yang 2020

Services and staffing practices in academic health sciences libraries serving college of osteopathic medicine programs: a mixed methods study

Joanne M. Muellenbach, AHIP; Wendy C. Duncan; Cheryl Vanier; Lisa A. Ennis; Anna Yang

APPENDIX B

Phase three: summarized responses to interview questions

The number of college of osteopathic (COM) library directors or leaders (out of seven interviewed) with a particular response is noted for each response.

1. Of the services that your library is offering, which services have had the most success in increasing the visibility and value of your library?

Service	n
Course director/teaching	2
Research project involvement	2
Physical use of library space*	2
Digital access	2
Digital marketing	1
Training in evidence-based medicine and information literacy	1
Services (poster printing)	1

* Physical use examples: reserved spaces, staff assistance, increased hours, and treadmill desks.

2. Could you explain the reasons for the success that you have experienced in integrating information literacy and evidence-based medicine (EBM) competencies in the medical school curriculum?

Reason	n
Student contact†	5
Position in teaching and service	3
Subject guides	1

† Student contact examples: one-on-one meetings, involvement in orientation, class instruction, workshops (with food), involvement in journal club, library staff helping as judges during research day presentations.

JMLA Journal of the Medical Library Association

SC

Supplemental content to
J Med Libr Assoc. Jul;108(3):dx.doi.org/10.5195/jmla.2020.862
www.jmla.mlanet.org
© Muellenbach, Duncan, Vanier, Ennis, Yang 2020

3. What do you think motivates you and your librarians to move beyond the traditional services and offer new services?

Motivation	n
Open to new activities and ideas	2
Requests from faculty and students	2
Campus event involvement	1
Evaluation structure	1
Library mission statement	1
Need to train and market services and resources	1
New and diverse personnel bringing new skills and ideas	1

4. Reviewing the responses to the services offered (in phase two), do you feel that you have sufficient staff (types and numbers)?

Response	n
Yes	2
No, we need 1 or 2 more librarians	2
No, we need to expand our services	2
No, we need an evening supervisor	1
No, we need to hire more tech-savvy personnel	1

5. Reviewing your responses to the services offered, what competencies and skills are needed by librarians and other professional staff to serve in these new roles?

Competency	n
EBM, study design, critical appraisal, biostatistics, and epidemiology	5
Data research management, research, and scholarly publishing	5
Interpersonal communication skills, building relationships outside library	5
Information technology (IT) infrastructure/networking skills	3
Systematic and scoping reviews	3
Accreditation process	1
Archives and special collections	1
Canvas and other course management software	1
Marketing and promotion of library services	1
Teaching or instruction, mapping objectives to medical competencies	1
Research guide development, web page creation	1
Strategic planning and time management	1
